# A new method for isolation and purification of fusion-competent inhibitory synaptic vesicles

**DOI:** 10.1016/j.crphys.2024.100121

**Published:** 2024-02-23

**Authors:** Nisha Gopal, Jeremy Leitz, Chuchu Wang, Luis Esquivies, Richard A. Pfuetzner, Axel T. Brunger

**Affiliations:** a Department of Molecular and Cellular Physiology, Stanford University, Stanford, USA; b Department of Neurology and Neurological Sciences, Stanford University, Stanford, USA; c Department of Structural Biology, Stanford University, Stanford, USA; d Department of Photon Science, Stanford University, Stanford, USA; e Howard Hughes Medical Institute, Stanford University, Stanford, USA

**Keywords:** Synaptic vesicle isolation, Fragment antigen-binding region (Fab), vGAT, Vesicular GABA transporter, Fusion assay

## Abstract

Synaptic vesicles specific to inhibitory GABA-releasing neurons are critical for regulating neuronal excitability. To study the specific molecular composition, architecture, and function of inhibitory synaptic vesicles, we have developed a new method to isolate and purify GABA synaptic vesicles from mouse brains. GABA synaptic vesicles were immunoisolated from mouse brain tissue using an engineered fragment antigen-binding region (Fab) against the vesicular GABA transporter (vGAT) and purified. Western blot analysis confirmed that the GABA synaptic vesicles were specifically enriched for vGAT and largely depleted of contaminants from other synaptic vesicle types, such as vesicular glutamate transporter (vGLUT1), and other cellular organelles. This degree of purity was achieved despite the relatively low abundance of vGAT vesicles compared to the total synaptic vesicle pool in mammalian brains. Cryo-electron microscopy images of these isolated GABA synaptic vesicles revealed intact morphology with circular shape and protruding proteinaceous densities. The GABA synaptic vesicles are functional, as assessed by a hybrid (*ex vivo/in vitro*) vesicle fusion assay, and they undergo synchronized fusion with synthetic plasma membrane mimic vesicles in response to Ca^2+^-triggering, but, as a negative control, not to Mg^2+^-triggering. Our immunoisolation method could also be applied to other types of vesicles.

## Highlights

• An engineered anti-vGAT Fab enables the specific isolation and purification of inhibitory (GABA) synaptic vesicles.
• Isolated GABA synaptic vesicles are structurally intact and have a circular shape, as assessed by electron microscopy.
• Isolated inhibitory synaptic vesicles are functional, as assessed by a single vesicle fusion assay.

## Graphical abstract

**Figure:**
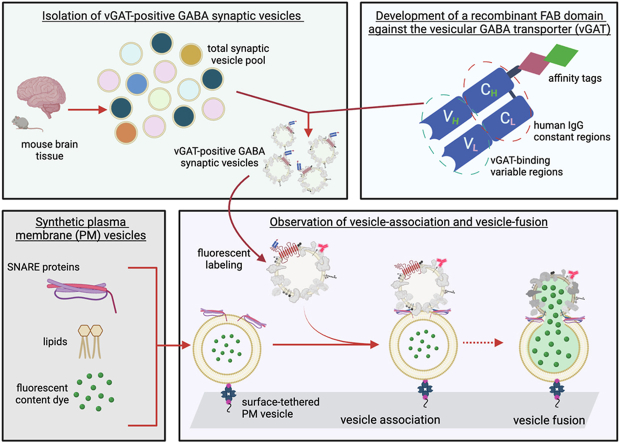

## 1 Introduction

Neurons form complex networks that contain both excitatory and inhibitory synaptic connections. In mature neurons, glutamate is the neurotransmitter predominating at excitatory synapses while GABA (γ-Aminobutyric acid) predominates at inhibitory synapses (Petroff, 2002). GABA-releasing neurons play an essential role in regulating the activity of all neuron types. The release of GABA molecules and subsequent binding to postsynaptic receptors causes a hyperpolarization of the postsynaptic membrane, which inhibits downstream action potential propagation (Krnjevic and Schwartz, 1967; Obata et al., 1967; Sears and Hewett, 2021). GABAergic interneurons have several critical modulatory functions in the mammalian central nervous system (reviewed in Fishell and Kepecs, 2020; Tremblay et al., 2016). Most importantly, they delimit the dynamic range of circuits to permit information transfer where under- or overexcitation might otherwise prevail. Thus, malfunction of these GABA-mediated regulatory activities can result in pathologies such as epilepsy, intellectual disability, autism spectrum disorder (Tang et al., 2021), major depressive disorder (Luscher et al., 2011), anxiety (Nuss, 2015), and schizophrenia (Costa et al., 2004), making this a critical therapeutic area.

The fusion of synaptic vesicles with the presynaptic plasma membrane is a key step for neurotransmission at chemical synapses, and the presynaptic fusion machinery consisting of SNAREs, the Ca^2+^ sensor synaptotagmin, and other proteins, is essential for this process (Brunger et al., 2018; Rizo and Xu, 2015; Rothman, 2014; Südhof, 2013). Synaptotagmin-1 and synaptotagmin-2 are found in isolated synaptic vesicles (Grønborg et al., 2010) and are Ca^2+^-sensors for fast synchronous release (Fernández-Chacón et al., 2001). Although the base fusion machinery is the same, the proteome of GABA SVs is somewhat distinct from that of glutamatergic (Glu) SVs. Specifically, the Ca^2+^ sensor synaptotagmin-1 is differentially enriched between the two vesicle types (Grønborg et al., 2010), with GABA SVs containing half as much synaptotagmin-1 as Glu SVs (Grønborg et al., 2010). Proteomic profiling revealed differences between excitatory and inhibitory synapses, including proteins that interact with voltage-gated calcium channels (VGCCs) (Loh et al., 2016). Morphologically, the spatial distribution of GABA and Glu SVs throughout their respective synapses may be different: the arrangement of VGCCs at synapse-specific active zones is different for inhibitory stellate cells and excitatory granule cells (Rebola et al., 2019). Cultured hippocampal neurons display morphological differences in the postsynaptic density between excitatory and inhibitory synapses (Tao et al., 2018). Thus, Ca^2+^ triggering could be differentially regulated in different synapses. Future investigations of molecular differences between GABA SVs and Glu SVs would benefit from methods to readily isolate and purify specific SVs.

Previous neurotransmitter-specific isolation of SVs used monoclonal antibodies against a particular subtype (Grønborg et al., 2010; Takamori et al., 2000). This method enabled the immunoprecipitation of specific SVs using IgG binding protein-G resin and was suitable for functional neurotransmitter uptake and proteomic analysis by mass spectrometry (Grønborg et al., 2010; Takamori et al., 2000). Subsequent elution of vesicles involved very low pH buffers and detergents (Grønborg et al., 2010; Takamori et al., 2000), and thus this method would not be suitable for studies of intact SVs. Moreover, when left bound to beads, contamination of the sample by non-specifically bound components could pose a challenge. As an alternative approach, a combined pool of SVs was purified by chromatography and then visually separated into specific SVs by labeling with subtype-specific fluorescent labels (Farsi et al., 2016). However, sorting of specific SVs based on the fluorescent label may not yield sufficient quantities for subsequent structural and functional studies. More recently, relatively pure and intact Glu SVs were obtained by affinity isolation using a monoclonal mouse anti-vGlut antibody, and elution with a peptide that corresponds to the antibody epitope (Brunger and Leitz, 2023, Leitz et al., 2024a, Leitz et al., 2024b). However, when we attempted a similar approach to immunoisolate and purify GABA SVs with anti-vGAT antibody (Synaptic Systems) we were unable to elute the GABA SVs from the antibody by using similar epitope-derived peptides. Therefore, we developed an alternative method to immunoisolate and purify intact GABA SVs.

Here we present a new method for the isolation of intact GABA SVs from mouse brain tissue via a fragment antigen-binding region (Fab) conjugated to a Twin-Strep tag. Rather than eluting the GABA SVs from Fab-conjugated beads, the Fab remains bound to the GABA SV, and the complex is eluted from the Streptactin resin with biotin. Our new method yields relatively pure and intact GABA SVs with circular shape and a tight size distribution. We further demonstrate that the isolated GABA SVs are functional as they fuse with plasma membrane (PM)-mimic vesicles upon triggering with Ca^2+^, but, as a negative control, not with Mg^2+^.

## 2 Materials and methods

### 2.1 Cloning, expression, & purification of an engineered anti-vGAT fab

**Fig. 1:**
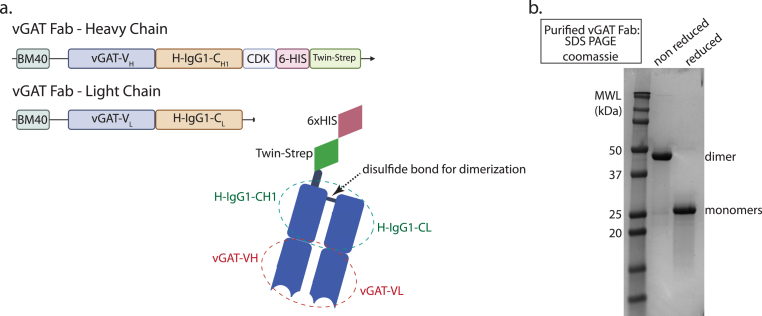
**Design, expression, and purification of an anti-vGAT Fab domain (a)** Expression plasmids encoding for the Fab heavy and light chains. Heavy chain and light chains contained a BM40 signal sequence which facilitated secretion of expressed protein into the cell culture media for easy purification. Both constructs included a variable region and constant region, and the heavy chain additionally included a CDK (Cysteine-Glutamate-Lysine) motif for Fab dimerization, and for affinity purification, a hexahistidine (6-HIS) tag followed by a Twin-Strep tag. Cartoon schematic (BioRender) of the engineered recombinant anti-vGAT Fab domain dimer used for isolation of native vGAT synaptic vesicles. **(b)** Coomassie-stained SDS PAGE analysis of the purified anti-vGAT Fab region.

The DNA sequences encoding anti-vGAT Fab fragments of heavy and light variable chains were generously provided by James Trimmer (Neuromab Core Facility at the University of California, Davis, https://neuromabseq.ucdavis.edu/). Human IgG constant region sequences were combined with the variable region sequences of a monoclonal antibody against vGAT to (Fig. 1a), simply referred to as vGAT Fab in the following. Heavy and light chain DNA sequences were synthesized by Integrated DNA Technologies (Coralville, IA) and cloned into gWIZ mammalian expression vectors containing human IgG heavy and light chain constant regions (generously provided by Jack Silberstein, Stanford University) using Gibson assembly. The heavy chain construct was conjugated to C-terminal 6xHis and TwinStrep tags, and both heavy and light chain constructs had N-terminal secretion signals for export into culture media. The vGAT Fab constructs were co-expressed in Expi293F HEK (Thermo Fisher Scientific, A14527) by co-transfecting the two constructs at a 1:1 ratio and culturing the cells in Expi293F growth media at 37 °C, 8% CO_2_. After five days, cells were centrifuged at 5000 RPM for 10 min, and the supernatant containing secreted vGAT Fab was collected and adjusted to 20 mM HEPES pH 7.4 and 300 mM NaCl. The supernatant was mixed with Ni-NTA resin pre-equilibrated with wash buffer (20 mM HEPES pH 7.4, 300 mM NaCl, 30 mM Imidazole), and incubated overnight at 4 °C with stirring. Bound vGAT Fab was eluted with wash buffer containing 450 mM Imidazole and further purified by size exclusion chromatography (SEC) in the presence of 20 mM HEPES pH 7.4 and 150 mM NaCl, using a Superdex 200 16/600 preparative grade column (Cytvia, 28989335). Peak fractions were pooled and the presence of the purified vGAT Fab at approximately 50 kDa was confirmed via SDS PAGE; the samples were then aliquoted and stored at −80 °C.

### 2.2 Immunoisolation and purification of GABA SVs

Synaptic vesicles were isolated following previously published procedures ;(Grønborg et al., 2010, Leitz et al., 2024a, Leitz et al., 2024b). Briefly, crude synaptic vesicles (lysis pellet 2 or “LP2”) were generated from freshly harvested mouse brain tissue from twelve 23–25 days old CD1 mice (Charles River) per preparation. 560 μL of 32 μM purified vGAT Fab was incubated with 33.3 μL bed volume of paramagnetic streptactin resin (IBA, 2-1613-002) pre-equilibrated with vesicle buffer (“VB”: 20 mM HEPES pH 7.4, 90 mM NaCl) for approximately 24 h. Streptactin resin was pelleted using a magnetic stand and the supernatant was discarded. vGAT Fab bound resin was then washed with 20 column volumes of VB supplemented with 0.2% Casein blocker (Thermo Fisher Scientific, PI37582). Washed and blocked vGAT Fab bound resin was incubated overnight with 1–2 mg of LP2 in 1.4 mL final volume. Flow-through was collected on the magnetic stand and beads were washed thoroughly with 6 mL of 0.2% Casein-VB. Beads were then resuspended in 500 μL and incubated with fluorescent anti-synaptophysin antibody (at a 1:500–1:8000 ratio) (Abcam, ab196166) at room temperature for 2–3 h.

Following fluorescent labeling, beads were washed with 10 column volumes of VB and then eluted with 35–50 μL VB supplemented with 50 mM biotin. 35–50 μL elution fractions were collected and analyzed by Western blot (Supplementary Table 1). SYP-labeled GABA SVs are typically eluted at fractions 4–7. These fractions were pooled and dialyzed against 1–2 L of VB supplemented with 20 μM ethylene glycol-bis (β-aminoethyl ether)-N, N, N′, N′-tetraacetic acid (EGTA) to remove excess biotin and free anti-SYP fluorescent antibody.

### 2.3 Preparation of recombinant, purified syntaxin-1A and SNAP-25A

Full-length rat syntaxin-1A was prepared as previously described (Diao et al., 2013, Lai et al., 2017, Leitz et al., 2024a, Leitz et al., 2024b). Cysteine-free SNAP-25A was expressed and purified as described previously (Lai et al., 2017, Leitz et al., 2024a, Leitz et al., 2024b).

### 2.4 Preparation of PM-mimic acceptor vesicles

PM-mimic acceptor vesicles with reconstituted syntaxin-1A and SNAP-25A were generated as described previously (Diao et al., 2013; Kyoung et al., 2013; Lai et al., 2017, Leitz et al., 2024a, Leitz et al., 2024b). Briefly, a lipid film of Porcine Total Brain Extract (Avanti Polar Lipids, 131101C), 3% PIP2 (Avanti Polar Lipids, 840046X), 1% Biotin PE (Avanti Polar Lipids, 870285P) and 1% DAG (Avanti Polar Lipids, 800811C) was generated by combining the lipids followed by evaporation under argon. Lipid films were allowed to dry overnight under a vacuum. PM vesicles were reconstituted with syntaxin-1A and SNAP-25A in a VB containing 50 mM of sulforhodamine B (Thermo Fisher Scientific). The protein-lipid mixture was then added to a 5-mL CL-4B (Millipore Sigma GE17-01501-01) column. 200 μL fractions were collected and analyzed on an SDS PAGE gel with Coomassie stain to assess which fractions had visible bands for both syntaxin-1A and SNAP25. Select fractions were pooled and dialyzed overnight at 4 °C against 2 L of VB with Bio-Beads SM-2 resin (BioRad, 1523920). Vesicle size and homogeneity were determined via dynamic light scattering (DLS) using a DynaPro NanoStar (Wyatt Technologies) (Supplementary Fig. 3).

### 2.5 Fusion assay data collection and analysis

Prism-based total internal reflection microscopy (TIRF) was performed on a Nikon Eclipse Ti–U with a 60*x* water immersion objective, as previously (Kyoung et al., 2013). Images were collected on an Andor iXon Ultra-897 EMCCD camera (Andor-Oxford Instruments) attached to the microscope via an Optosplit I (Technical Instruments). Quartz slides were prepared as described previously (Leitz et al., 2023a). Briefly, Neutravidin (Thermo Fisher Scientific) was added at 0.1 mg/mL and incubated for 5 min followed by washing with ∼50 vol (200 μL) of VB. PM vesicles were diluted 20–50*x* in VB then added to flow cell chambers and allowed to incubate for 5 min. Unbound PM vesicles were removed by washing the flow cell with ∼50 vol of vesicle buffer. Slides were then mounted to the microscope. Anti-Synaptophysin-Alexa 647-labeled SVs were injected, and vesicle association was monitored for 1 min. After image acquisition, unbound SVs were again washed away with ∼50 vol of VB. Image acquisition resumed after 30 frames of baseline, Ca^2+^ or Mg^2+^ at the reported concentrations in VB containing free Alexa-647 dye to monitor the arrival of the injected solution. Image acquisition lasted for 1 min. Films were then separated as either “association” or “fusion” films for subsequent analysis.

Single vesicle fusion data were analyzed using custom Matlab (Mathworks) scripts, as previously described (Lai et al., 2017, Leitz et al., 2024a, Leitz et al., 2024b). However, at variance to (Leitz et al., 2024a, Leitz et al., 2024b), in this study, it was not possible to accurately determine the associated GABA SV-PM pair number due to only partial labeling efficiency of GABA SVs (Supplementary Fig. 3). Therefore, we could not normalize the histograms of fusion events to the number of associated GABA SV-PM vesicle pairs, and we could not assess the fusion efficiency of the GABA SVs. However, we could quantify the fusion synchronization since this quantity does not depend on the number of associated GABA SV-PM vesicle pairs. We assessed fusion synchronization by two different methods. (1) We calculated the fraction of fusion events in the first second (∼5 acquisition frames) of all fusion events observed during the entire acquisition period after Ca^2+^ injection. (2) We fit the histograms of fusion events to two-phase exponential decay functions using nonlinear regression with GraphPad (GraphPad Software).

### 2.6 SDS PAGE and western blotting

GABA SV isolation fractions were analyzed via gel electrophoresis for total protein staining and Western blot analysis. Membranes were subsequently incubated in a combination of primary antibodies in a Western blot blocking buffer. Washes were done with 1× PBS after antibody incubation, and secondary antibody incubation involved a combination of secondary antibodies in a Western blot blocking buffer. See Supplementary Table 1 for full details.

### 2.7 Negative stain electron microscopy, immunogold labeling

Copper carbon electron microscopy grids (EMS, CF300-CU-50) were glow discharged at 15 mA for 30 s using a Pelco easiGlow system (TED PELLA, Inc.). 5 μL of sample was applied to the grid surface and incubated for 5 min. Grids were blotted and stained with 1.0% uranyl acetate solution and imaged on a JEM1400 120 kV transmission electron microscope equipped with an Orius CCD camera (Gatan). For immunogold labeling experiments, 5 μL of GABA SV sample was applied to copper carbon electron microscopy grids. After sample application and blotting, grids were blocked in VB supplemented with 0.2% casein for 10 min at room temperature. Then, grids were blotted and incubated in a 1:10 solution of 15 nm gold anti-rabbit (Cytodiagnostics, AC-15-06-05) Fab for 15 min at room temperature. Finally, grids were blotted, washed with a vesicle buffer, and stained with 1.0% uranyl acetate solution.

### 2.8 Cryo-electron microscopy (Cryo-EM)

Fractions from GABA SVs isolations were pooled, and 6.5 μL of the sample was applied twice to Quantifoil R1/4 or R1/2 holey carbon-gold mesh EM grids, first with 3.5 μL for at least 8 min at room temperature in a humidity chamber and again with 3.0 μL on a Vitrobot Mark IV (Thermo Fisher Scientific) with a wait time of 10 s. Grids were back blotted with standard Grade 595 Vitrobot filter paper (Whatman) on the back side and parafilm (BEMIS PM996) on the sample side for 4 s at 22 °C, with a blot force of 5 at 100% humidity, followed by rapid plunging into liquid ethane. Images were collected using SerialEM (Mastronarde, 2005) on a Glacios transmission electron microscope (Thermo Fisher Scientific, USA) at 200 kV equipped with a K3 direct electron detector (Gatan) and a Titan Krios (Thermo Fisher Scientific, USA) at 300 kV equipped with a K3 direct electron detector. Four sets of dose fractionated movies were collected (three in normal mode and one in super-resolution mode) with physical pixel sizes of 2.43, 2.18, 2.34, and 1.11 Å/pixel, and a digital pixel size of 0.55 Å/pixel for the movies collected in super resolution mode. Movies were collected on the Glacios and Krios microscopes. Cryo-EM micrographs of GABA SVs were corrected for beam-induced motion using MotionCorr2 (Li et al., 2013). Diameters were calculated from area using ImageJ (https://imagej.net/ij/index.html) by manually drawing around the perimeters of ten chosen vesicles per micrograph, subsequently measuring the vesicle area (using the appropriate pixel size for each micrograph), and then calculating the diameter using the formula for the area of a circle. For these diameter measurements, a total of 100 vesicles were chosen by randomly selecting 10 vesicles each in 10 randomly selected micrographs (of a total of 50 micrographs, see Data Repository), across two of four datasets, each representing independent preparations of LP2 and GABA SVs, excluding particles that were abnormally large or small.

## 3 Results

### 3.1 Immunoisolation and purification of GABA SVs

As mentioned above, initial attempts to immunoisolate GABA SVs from mouse brain tissue using a monoclonal antibody against vGAT (Synaptic Systems) failed as we could not achieve peptide-based dissociation between monoclonal vGAT antibodies and bound GABA SVs, perhaps due to a higher affinity of the antibody and the vesicular transporter. Therefore, we developed a recombinantly engineered Fab (Trimmer, 2020) with a human IgG constant region combined with the variable region sequences of a monoclonal antibody against vGAT and conjugated to a twin-strep tag (Fig. 1a). The interaction between the twin-strep tag and resin-bound streptactin is disrupted by the addition of biotin. This method facilitates the dissociation of Fab-bound GABA vesicles from the resin.

**Fig. 2:**
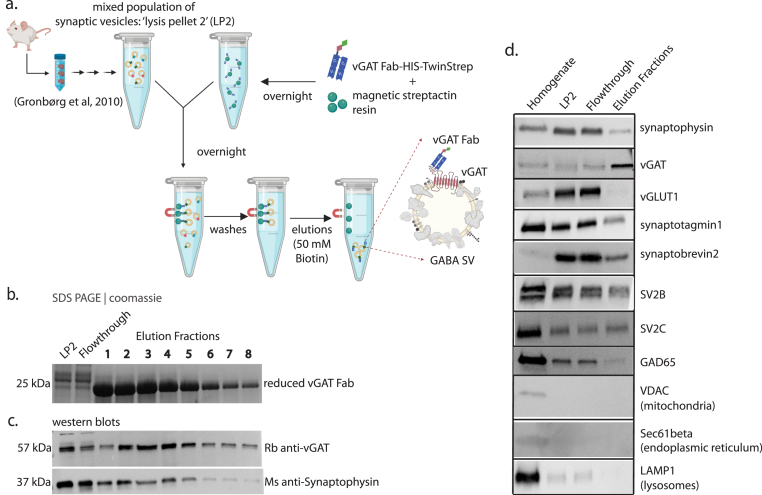
**Method for isolation of GABA SVs. (a)** Cartoon schematic (BioRender) of GABA SV isolation method. **(b)** Coomassie-stained SDS PAGE analysis of GABA SV isolation fractions including starting material LP2, flow through, and elution fractions 1–8. (**c**) Western blot analysis of GABA SV isolation fractions (same samples as in panel b). Top: Western blot using a rabbit polyclonal antibody against the luminal region of vGAT. Bottom: Western blot using a mouse polyclonal antibody against synaptophysin. Both proteins were co-blotted on the same membrane. **(d)** Western blots of mouse brain homogenate, LP2, flowthrough, and elution fractions. While all samples were loaded at equal volume, they were not loaded at equal protein amount or concentration. Antibody information is provided in Supplementary Table 1.

We tested whether the purified vGAT Fab could be used to immunoisolate GABA SVs from a total synaptic vesicle population prepared from mouse brain tissue using established protocols (Grønborg et al., 2010; Takamori et al., 2000) (Fig. 2a). The vGAT Fab was secreted into cell culture media after expression, greatly simplifying purification. This approach produced purified vGAT Fab (∼50 kDa) (Fig. 1b) with an average protein yield of 50 mg/L of cell culture. The total synaptic vesicle population was combined with vGAT Fab-saturated paramagnetic streptactin beads to tether the GABA SVs to the resin (Fig. 2a top). GABA SVs with bound vGAT Fab were eluted from the streptactin resin using biotin (Fig. 2a bottom). Upon addition of 50 mM biotin, 35 μL fractions were collected, as expected, vGAT Fab eluted immediately (Fig. 2b and Supplementary Fig. 1b). We probed for vGAT and synaptophysin (SYP), a ubiquitous SV protein using western blotting (Fig. 2c and Supplementary Fig. 1a) and observed the majority of protein eluting in fractions 1–8 (280 μL total elution volume), with the band intensity typically peaking between elution fractions 2 and 5 (Fig. 2c and Supplementary Fig. 1a). To assess the specificity of the elution we probed for the vesicular glutamate transporter (vGLUT1), and as expected, vGLUT1 was largely depleted from the elution fractions while being present in all other steps including flowthrough (Fig. 2d and Supplementary Fig. 1c). Next, we confirmed the presence of other expected SV proteins including synaptobrevin2, synaptotagmin1, SV2B, SV2C and GAD65 (Fig. 2d and Supplementary Fig. 1c). Moreover, we confirmed that the GABA SV sample was largely free of cellular contaminants such as mitochondria, lysosomes, and endoplasmic reticulum by western blotting for the proteins: voltage-dependent anion channel (VDAC), lysosome-associated membrane protein 1 (LAMP1), and Sec61b, respectively (Fig. 2d and Supplementary Fig. 1c).

### 3.2 Isolated and purified GABA SVs are morphologically intact

**Fig. 3:**
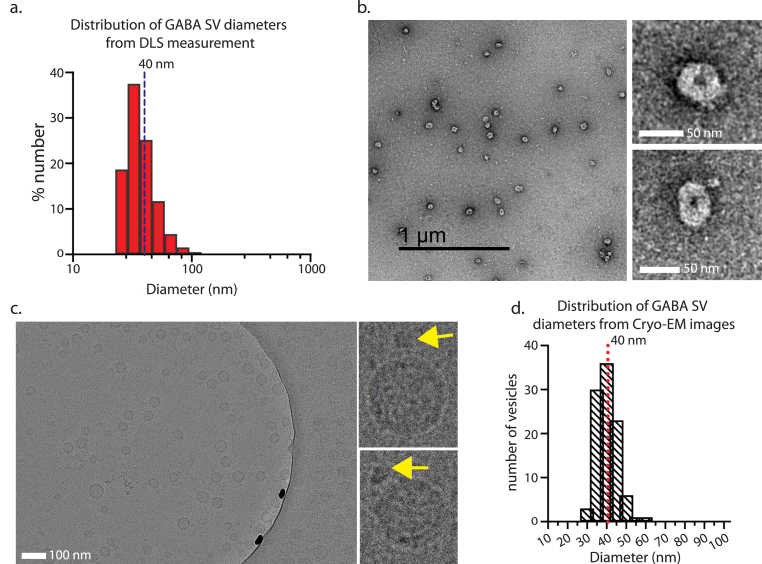
**Electron microscopy imaging of GABA SVs. (a)** Representative dynamic light scattering (DLS) measurement of purified GABA SVs plotted as a frequency distribution of measured particle diameters. n = 3 SV isolations, three DLS measurements. **(b)** Representative negative stain micrograph of purified GABA SVs. On the right, two magnified view examples of individual GABA SVs were taken from different representative micrographs. **(c)** Representative cryo-EM micrograph of isolated GABA SVs, with a physical pixel size of 0.243 nm/pixel. Cropped magnified views of individual GABA SVs from cryo-EM micrographs, with yellow arrows pointing to large visible protein densities, likely the v-ATPase. **(d)** Histogram displaying the frequency distribution of GABA SV diameters measured from cryo-EM micrographs using ImageJ; the dotted line represents the mean diameter value of 40.4 ± 0.5 nm. n = 2 isolations, 10 micrographs, 100 measured vesicles. All micrographs are available in the data repository associated with this work. (For interpretation of the references to color in this figure legend, the reader is referred to the Web version of this article.)

The size distribution of the purified GABA SVs was measured by dynamic light scattering (DLS). Vesicle diameters range from ∼30 nm to ∼80 nm, with a geometric average of 43.7 ± 4.42 nm (Fig. 3a). Negative stain EM images revealed circular vesicle-like particles of uniform shape (Fig. 3b), consistent with previous findings (Takamori et al., 2000). Since negative stain EM often produces staining artifacts and membrane distortions of vesicular samples, we also used cryo-EM imaging (Fig. 3c). Cryo-EM micrographs of GABA SVs revealed intact, circular vesicles with an average SV diameter of 40.4 ± 0.50 nm (Fig. 3c, d, and 84 deposited Cryo-EM micrographs). The observed mean diameter is consistent with the DLS measurements, although the variation is smaller presumably since the DLS measurements represent an average of all particles, including some unusually large and small particles. Circular vesicles were observed in all micrographs except for one EM grid where several oval vesicles were observed. The blotting and freezing conditions may likely have caused this phenomenon for this particular grid, for example, very thin ice may have deformed the vesicles. Thus, we excluded the micrographs collected from this particular grid from the analysis. We observed visible proteinaceous densities (yellow arrows, Fig. 3c) protruding from some of the GABA SV membranes in the cryo-EM images. The most likely candidate for the largest protruding density is the V1 subunit of the V-ATPase, consistent with previous studies which showed that SVs typically contain 1 to 2 copies of the V-ATPase complex per SV (Takamori et al., 2006). Taken together, our isolation and purification method produced morphologically intact SVs with a relatively tight size distribution.

### 3.3 GABA SVs associate with PM acceptor vesicles

**Fig. 4:**
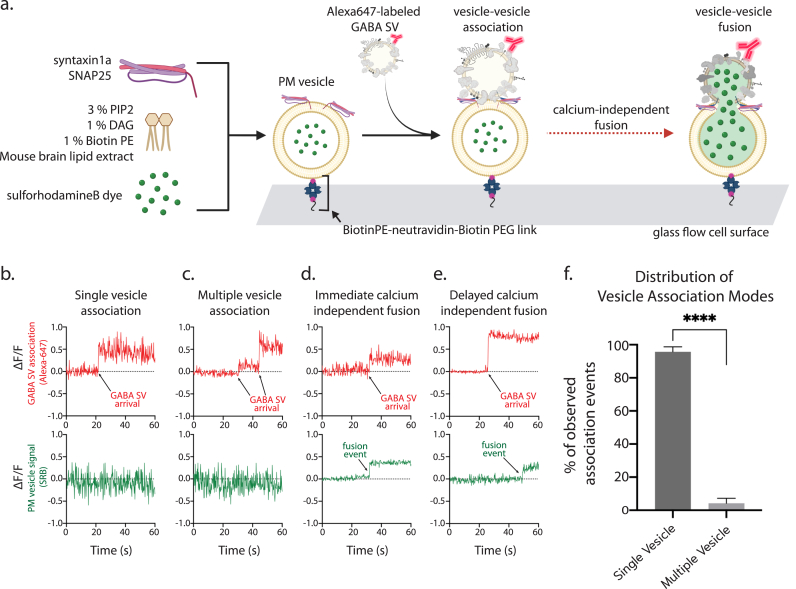
**Association of GABA SVs with PM vesicles. (a)** Cartoon schematic (BioRender) of the GABA SV-PM association assay. **(b**–**e)** Representative examples of individual association events, plotting baseline-normalized fluorescence vs. time, the red traces report the fluorescence due to the Alexa-647 dye present on the GABA SVs while green traces report the sulforhodamine B fluorescence inside the PM vesicles. See Supplementary Table 2 for details. **(f)** Bar graph detailing the relative distribution of association types observed in the total collected dataset of association movies. n = 3 technical replicates, 25 movies, 9728 p.m. vesicles, 320 association events. Number of Ca^2+^-independent fusion events was 3 out of 296 single vesicle association events. Shown are the mean and standard error of the mean, and the student's *t*-test was used to test statistical significance. All association data are available in the data repository associated with this work. (For interpretation of the references to color in this figure legend, the reader is referred to the Web version of this article.)

Next, we tested whether the GABA SV samples associate and fuse with target membranes. We used a hybrid (*ex vivo/in vitro*) single vesicle fusion assay (Leitz et al., 2023a) to monitor the association between GABA SVs and PM-mimic acceptor vesicles (simply referred to as PM vesicles in the following) (Fig. 4a). Briefly, PM vesicles were first tethered to a flow chamber by PEG-biotin-Neutravidin-BiotinPE, followed by several washes to remove unbound PM vesicles (Fig. 4a). Alexa-647-labeled GABA SVs then were injected into the flow cell (Fig. 4a). We categorized the observed association events into “single vesicle association” and “multiple vesicle association” events. Single GABA SV-PM vesicle association events were characterized by a single stepwise increase in Alexa-647 fluorescence intensity (Fig. 4b), whereas multiple vesicle association events were characterized by multiple stepwise increases in Alexa-647 fluorescence intensity (Fig. 4c). Vesicle association was overwhelmingly composed of single association events of labeled vesicles (consistent with Leitz et al., 2024). Approximately 95.8 ± 2.98% of the association events contained single vGAT SV-PM vesicle associations, whereas there were only 4.2 ± 2.98% of labeled vesicle association events involved multiple associations (Fig. 4f). We note that we only achieved partial labeling efficiency of the GABA SVs (Supplementary Fig. 3); most likely this is due to a relatively lower number of synaptophysin (SYP) molecules in GABA compared to Glu SVs (Grønborg et al., 2010) (note that we achieved much higher labeling efficiency of Glu SVs, see refs (Leitz et al., 2024a, Leitz et al., 2024b) , and a relatively low concentration of the GABA SVs that precluded using a saturating concentration of the SYP antibody since otherwise many unbound labeled SYP-antibodies would obscure the signal from specifically docked GABA SVs in our fusion experiments. Therefore, there will be some undercounting of association events, although the results suggest that multiple association events are relatively rare. Of the total number of GABA SV-PM vesicle association events observed, 320 total events, only three associations proceeded to fusion in the absence of Ca^2+^. Of those three that did undergo fusion, one pair did so immediately upon vesicle association (Fig. 4d), while the other two had a dwell time (the time between formation of an associated pair and fusion) of 12.7 and 23.6 s, respectively (Fig. 4e).

### 3.4 Ca^2+^-triggered fusion of GABA SVs and PM vesicles

**Fig. 5:**
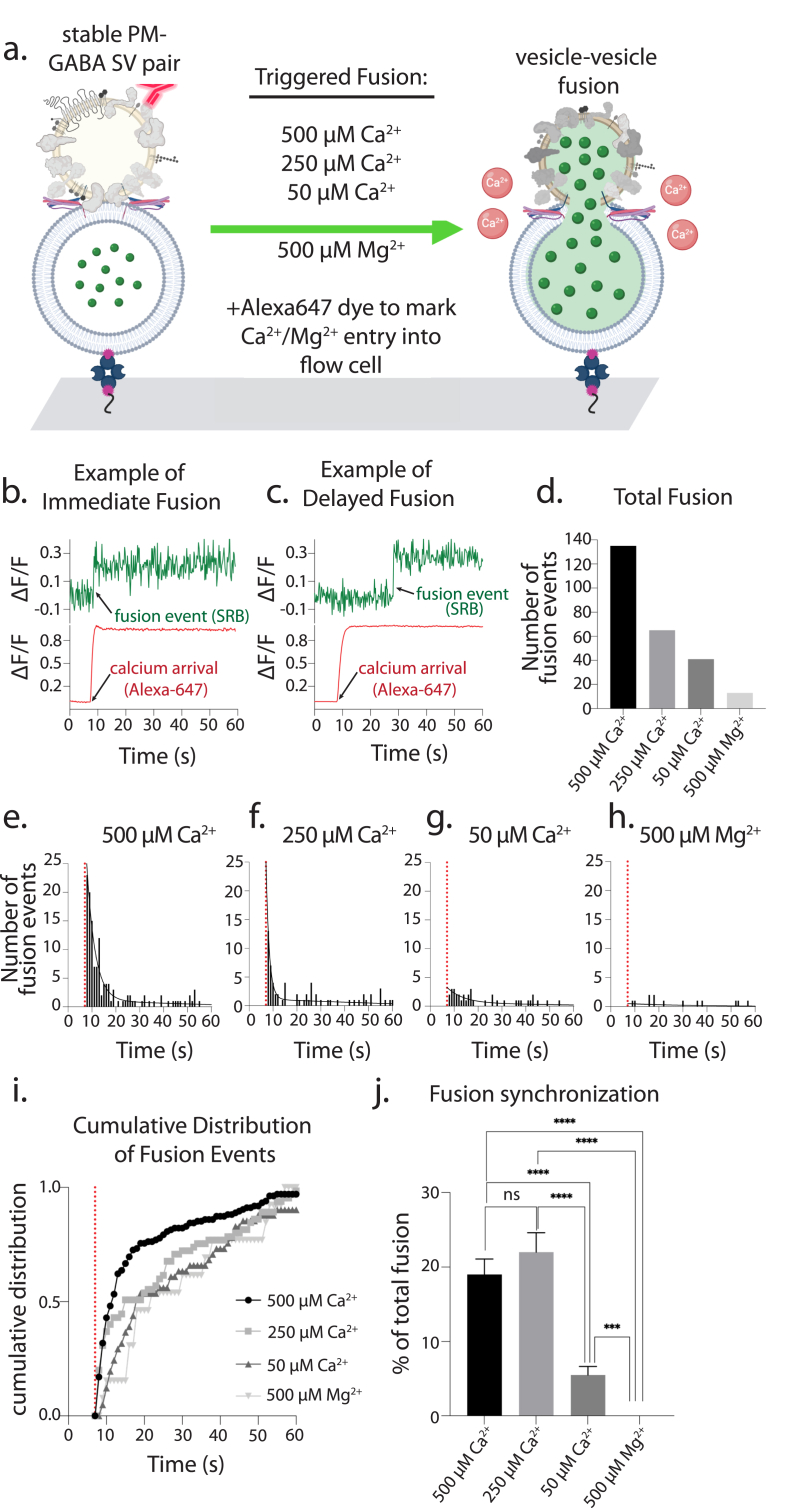
**Triggered fusion between GABA SVs and PM vesicles. (a)** Cartoon schematic of fusion triggering experiments in the hybrid *in vitro* system. **(b)** A representative example of Ca^2+^-triggered immediate fusion at 500 μM Ca^2+^ concentration, with the green trace representing SRB fluorescence intensity (PM vesicle) and the red trace representing Alexa-647 fluorescence intensity (Ca^2+^ entry). **(c)** A representative example of Ca^2+^-triggered delayed fusion at 500 μM Ca^2+^ concentration (same color scheme as in b). **(d)** Bar graph plotting the total number of observed fusion events for each Ca^2+^ and Mg^2+^ condition tested. See Supplementary Table 3 for details. **(e**–**h)** Frequency distribution histograms of fusion event times (all normalized to a Ca^2+^/Mg^2+^ injection time of 7 s). Each histogram was fit to a two-phase exponential decay function using GraphPad with the following R Squared values: 0.927 for 500 μM Ca^2+^, 0.811 for 250 μM Ca^2+^, 0.542 for 50 μM Ca^2+^, and 0.114 for 500 μM Mg^2+^. **(i)** Cumulative distribution plot of fusion event times for each condition. **(j)** Fusion synchronization for each Ca^2+^/Mg^2+^ condition calculated as the number of fusion events in the first second (5 acquisition frames) after triggering (statistical details are in Supplementary Table 3). Due to the small number of fusion events for the low Ca^2+^ concentration condition and the Mg^2+^ condition, determining the statistical significance on a per repeat-experiment basis is not reliable. We therefore used bootstrapping methods (one with data replacement and one without data replacement) to determine the mean, standard deviation, and statistical significance. Both bootstrapping methods produced very similar results. Shown is the bootstrapping analysis without replacement, with 10 random subsets extracted from each condition with a subset size of 20 “data items” (number of fusion events in the first second) for 500 μM Ca^2+^, 250 μM Ca^2+^, and 50 μM Ca^2+^, and 10 data items for 500 μM Mg^2+^. The mean of the 10 subsets for each condition is plotted as a bar graph and the error bars are standard errors of the mean. Unpaired, two-tailed t-tests were done to compare fusion synchronization between 500 μM Ca^2+^ vs. 250 μM Ca^2+^ (P = 0.3800), 500 μM Ca^2+^ vs. 50 μM Ca^2+^ (P < 0.0001), 250 μM Ca^2+^ vs. 50 μM Ca^2+^ (P < 0.0001), 500 μM Ca^2+^ vs. 500 μM Mg^2+^ (P < 0.0001), 250 μM Ca^2+^ vs. 500 μM Mg^2+^ (P < 0.0001), and 50 μM Ca^2+^ vs. 500 μM Mg^2+^ (P = 0.0002). All fusion data are available in the data repository associated with this work. (For interpretation of the references to color in this figure legend, the reader is referred to the Web version of this article.)

We next tested the Ca^2+^-triggered fusion capability of the GABA SV–PM vesicle pairs by injecting Ca^2+^, and as a negative control, Mg^2+^ (Fig. 5a). We note that GABA SVs contain synaptotagmins (Grønborg et al., 2010), including the Ca^2+^ sensors for fast synchronous release, synaptotagmin-1 and synaptotagmin-2. We observed fusion events both concomitant with Ca^2+^ arrival (Fig. 5b) or delayed after Ca^2+^ arrival (Fig. 5c), similar to previous experiments with Glu SVs (Lai et al., 2017, Leitz et al., 2024a). As expected, decreasing the Ca^2+^ concentration decreased the number of fusion events that were observed, with 500 μM Ca^2+^ triggering leading to 135 observed fusion events, 250 μM Ca^2+^ leading to 65 fusion events, and 50 μM Ca^2+^ leading to only 41 fusion events (Fig. 5d) (Supplementary Table 3). Importantly, the negative control experiment with 500 μM Mg^2+^ produced only 13 fusion events (Fig. 5d).

Next, we measured the time between Ca^2+^ arrival and fusion. We again observed a strong Ca^2+^-concentration dependence in the latency between fusion and Ca^2+^ arrival (Fig. 5e–j). For high (500 μM) Ca^2+^ triggering, many fusion events occurred immediately upon arrival of Ca^2+^ followed by a rapid decay in fusion frequency, similar to previous experiments with wholly synthetic SV system (Lai et al., 2017) or Glu SVs (Leitz et al., 2023a). Interestingly, the decay in fusion was not well fit with a single exponential decay but required a two-phase exponential fit (Fig. 5e–j, black lines), at variance to a fully synthetic system (Diao et al., 2012; Lai et al., 2014, 2017). This observation is consistent with the bi-phasic observed fusion decay using Glu SVs and a similar reconstitution method with PM vesicles (Leitz et al., 2023a). 500 μM Ca^2+^ triggered fusion decayed with 70% of the fit having a fast decay constant of 0.3 s^−1^ and 30% of the fit containing a slow decay constant of 0.001 s^−1^ (Fig. 5e), compared to 250 μM Ca^2+^ for which 88% of the fit having a fast decay constant of 0.7 s^−1^ and 12% of the fit containing a slow decay constant of 0.005 s^−1^ (Fig. 5f). 50 μM Ca^2+^ only minimally elicited fusion with a nearly flat decay with 31% of the fit corresponding to a decay constant of 0.1 s^−1^ and 70% of the fit corresponding to a decay constant of 0.001 s^−1^ (Fig. 5g). In contrast, as a negative control, 500 μM Mg^2+^ elicited very desynchronized fusion with 36% of the fit corresponding to a decay constant of 0.05 s^−1^ and 64% of the fit corresponding to a decay constant of 0.007 s^−1^ (Fig. 5h).

The cumulative probability distributions show that for 500 μM Ca^2+^ triggering, more than 50% of fusion events occurred within ∼5 s after Ca^2+^ injection, whereas for 250 μM Ca^2+^ triggering, it took ∼8 s to reach 50% (Fig. 5i). Triggering by 50 μM Ca^2+^ took about 11 s to achieve 50% fusion (Fig. 5i) as did triggering by 500 μM Mg^2+^, although we note in the case of Mg^2+^ there were so few events, and they appear to be largely stochastic events (Fig. 5h). We next calculated a fusion synchronization, defined as the amount of fusion occurring in the first second (5 acquisition frames) after Ca^2+^/Mg^2+^ arrival normalized to the total amount of fusion events observed during the entire acquisition period (Supplementary Table 3). Within the first second after Ca^2^ arrival, 19.0 ± 2.1% fusion events were triggered by 500 μM Ca^2+,^ and 22.0 ± 2.6% fusion events were triggered by 250 μM Ca^2+^ (Fig. 5j). 50 μM Ca^2+^ led to only 5.5 ± 1.2% fusion events within the first second after Ca^2+^ arrival. In contrast, and as expected, 500 μM Mg^2+^ did not result in any fusion events within that same time frame (Fig. 5j). Taken together, at the two higher Ca^2+^ concentrations tested, triggered fusion is dominated by a fast synchronous process, whereas at the low Ca^2+^-concentration, it is dominated by a slow process for the fusion assay with PM vesicles used in this work.

## 4 Discussion

Enrichment of intact GABAergic SVs from brain tissue presents a challenge, as GABAergic neurons make up a minority population of neuronal cells, that varies between brain regions. Of the total SV pool, GABA SVs only constitute approximately 15% of the total SV pool (Takamori et al., 2000; Upmanyu et al., 2022). In many prior studies, where SV fusion could be monitored, SVs were purified as a mixed population. Studies with isolated subsets of GABA SVs used antibodies that enabled quantitative proteomic comparisons (Grønborg et al., 2010), and functional assessment of transporter activities (Takamori et al., 2000), but intact GABA SVs could not be eluted. In these studies, isolated SVs were either left physically restrained to the resin matrix (used to purify them), or SVs were harshly processed for subsequent proteomic analysis by mass spectrometry. In more recent studies, post hoc identification using fluorescent markers based on the subtype (Farsi et al., 2016) or expression of exogenous proteins permitted either immunoprecipitation of SVs from cultured neurons via an HA-tag cloned onto the c-terminal end of synaptophysin (Chantranupong et al., 2020). More recently, we developed a method to isolate and purify intact Glu SVs by affinity isolation and elution with a peptide corresponding to the antibody epitope (Brunger and Leitz, 2023, Leitz et al., 2024a, Leitz et al., 2024b). However, we were unable to apply this approach to GABA SVs since the dissociation of GABA SVs from monoclonal antibodies via a peptide was unsuccessful. Therefore, we developed an alternative method to immunoisolate and purify intact GABA SVs. We engineered a recombinant Fab against vGAT (Fig. 1a). This approach offers several advantages: first, vGAT Fab can be expressed and purified at high yield and stored stably while retaining the ability to isolate GABA SVs. Second, the vGAT Fab can be conjugated to epitope tags to facilitate the biochemical isolation process.

Previous studies of isolated Glu SVs revealed primarily circular shapes with relatively narrow diameter distributions (Leitz et al., 2024a; Takamori et al., 2006). Here we find that the morphology of GABA SVs is also uniform with circular shapes and narrow diameter distributions (Fig. 3c and d). In contrast to the study by Tao et al. (2018), we do not find evidence of oval shapes except in a condition where freezing and blotting conditions may have deformed the synaptic vesicles. We also observe large densities on some of the vesicles that fit the profile of the V-ATPase, with only one or two per vesicle, as expected (Takamori et al., 2006).

We tested the function of the isolated and purified GABA SVs using a hybrid (*ex vivo*/*in vitro*) fusion system with PM vesicles (Fig. 4, Fig. 5), indicating that our isolation and purification method did not perturb the ability of the isolated GABA synaptic vesicles to undergo Ca^2+^-triggered fusion with the target membrane. GABA SV association occurred mostly as stable single vesicle-vesicle associations with very few association events involving multiple GABA SVs to the same PM vesicle. Importantly, very few Ca^2+^-independent fusion events occurred during vesicle association. When Ca^2+^ was injected, we observed triggered fusion events between vGAT SV and PM vesicles, with substantial synchronization at the higher Ca^2+^ concentrations. These results are similar to what has been previously reported for Glu SVs (Brunger and Leitz, 2023, Leitz et al., 2024a, Leitz et al., 2024b). Going forward it will be important to more closely study the kinetics of fusion between GABA and Glu SVs. However, the fusion assay presented here is a relatively simple system using PM vesicles that lacks accessory proteins, Munc13, Munc18, and complexin (Lai et al., 2017) as well as the disassembly machinery, NSF, and α-SNAP. As a next step, incorporating these additional proteins into our fusion assay will lead to a more robust and physiologically relevant fusion.

In closing, with the availability of isolated and purified GABA SVs, in combination with the controlled environment offered by a hybrid *in vitro* assay (Leitz et al., 2024b), we provide a useful platform for the study of therapeutics which are specific to GABA SV 1% of the entire human population is afflicted with epilepsy (a disorder thought to be caused by malfunction of GABAergic signaling), and 30–40% of those patients do not respond to current treatments which mainly target post-synaptic activity (Van Liefferinge et al., 2013), thus our platform represents an alternative means to test the presynaptic side of neuronal signaling. Moreover, although GABA release and basal GABA concentration regulation are obvious therapeutic target areas (e.g. Tiagabine and Vigabatrin, (Fueta et al., 2005)), a lack of mechanistic information regarding GABA release currently precludes more targeted approaches. Moreover, other proteins related to the synaptic machinery that are preferential to inhibitory neurons might be promising targets. SV2-family proteins may also be interesting targets given their differential expression in excitatory vs inhibitory neurons, particularly SV2C (Grønborg et al., 2010), and dopaminergic neurons (Dunn et al., 2017), given the anti-seizure drugs Levetiracetam and brivaracetam which target SV2A. Ultimately, there has been a gap in the development of tools needed to isolate and examine intact and functional GABA SVs toward these aforementioned goals, and now our isolation method can help to fill this gap.

## 5 Quantification and statistical analysis

The fusion experiments were conducted at least three times with different protein preparations and vesicle reconstitutions (details in Figure captions and Supplementary Tables 1 and 2), and properties were calculated as the mean ± s. e.m. Two-tailed Student's *t*-tests were used to test statistical significance in Fig. 4, Fig. 5j concerning the specified reference experiment. A bootstrap method was used in Fig. 5j (see figure caption).

## 6 Software and code

The smCamera software (Taekjip Ha laboratory, Johns Hopkins University, Baltimore) was used for acquisition. MATLAB (Mathworks) and Excel (Microsoft) were used to generate all curves and graphs. Custom MATLAB scripts were used for the analysis of the single-vesicle fusion experiments (see code availability). ImageJ (https://imagej.net/ij/index.html) was used for image analysis. BioRender was used for schematics. Grammarly was used to check for style and grammar at the final stage of revision.

## 7 Code availability

Custom MATLAB analysis scripts for the single-vesicle fusion experiments are available on GitHub https://github.com/brungerlab/single_molecule_matlab_scripts.

## Funding

This research was supported by the 10.13039/100000002National Institutes of Health for support (RO1MH63105 to A.T.B) and a grant by the Stanford ADRC Zaffaroni Alzheimer's Disease Translational Research Program to A.T.B. and J.L. (ADRC grant #5P50AG047366-02). The negative stain electron microscope was provided by the Stanford Cell Sciences Imaging Facility (NIH ORIP: 1 S10 OD028536-01).

## CRediT authorship contribution statement

Nisha Gopal designed, cloned, expressed, and purified the anti-vGAT Fab fragment, performed LP2 preparations, vesicle isolations, biochemical experiments, fusion experiments, data analysis, and wrote the paper. Chuchu Wang performed LP2 preparations. Luis Esquivies purified proteins used in the hybrid fusion assay. Richard Pfuetzner performed Fab purifications. Jeremy Leitz developed the hybrid fusion assay and custom MATLAB software to analyze the single vesicle fusion data. Axel Brunger designed experiments and wrote the paper.

## Data Availability

All raw data are available in the Stanford Digital Repository: https://doi.org/10.25740/ht023wd6382. PURL: https://purl.stanford.edu/ht023wd6382.

## References

[bib1] Brunger A.T., Choi U.B., Lai Y., Leitz J., Zhou Q. (2018). Molecular mechanisms of fast neurotransmitter release. Annu. Rev. Biophys..

[bib2] Brunger A.T., Leitz J. (2023). The Core complex of the Ca2+-triggered presynaptic fusion machinery. J. Mol. Biol., Protein-Membrane Interaction at the Pre-Synapse.

[bib3] Chantranupong L., Saulnier J.L., Wang W., Jones D.R., Pacold M.E., Sabatini B.L. (2020). Rapid purification and metabolomic profiling of synaptic vesicles from mammalian brain. Elife.

[bib4] Costa E., Davis J.M., Dong E., Grayson D.R., Guidotti A., Tremolizzo L., Veldic M. (2004). A GABAergic cortical deficit dominates schizophrenia pathophysiology. Crit. Rev. Neurobiol..

[bib5] Diao J., Grob P., Cipriano D.J., Kyoung M., Zhang Y., Shah S., Nguyen A., Padolina M., Srivastava A., Vrljic M., Shah A., Nogales E., Chu S., Brunger A.T. (2012). Synaptic proteins promote calcium-triggered fast transition from point contact to full fusion. Elife.

[bib6] Diao J., Zhao M., Zhang Y., Kyoung M., Brunger A.T. (2013). Studying protein-reconstituted proteoliposome fusion with content indicators in vitro. Bioessays.

[bib7] Dunn A.R., Stout K.A., Ozawa M., Lohr K.M., Hoffman C.A., Bernstein A.I., Li Y., Wang M., Sgobio C., Sastry N., Cai H., Caudle W.M., Miller G.W. (2017). Synaptic vesicle glycoprotein 2C (SV2C) modulates dopamine release and is disrupted in Parkinson disease. Proc. Natl. Acad. Sci..

[bib8] Farsi Z., Preobraschenski J., van den Bogaart G., Riedel D., Jahn R., Woehler A. (2016). Single-vesicle imaging reveals different transport mechanisms between glutamatergic and GABAergic vesicles. Science.

[bib9] Fernández-Chacón R., Königstorfer A., Gerber S.H., García J., Matos M.F., Stevens C.F., Brose N., Rizo J., Rosenmund C., Südhof T.C. (2001). Synaptotagmin I functions as a calcium regulator of release probability. Nature.

[bib10] Fishell G., Kepecs A. (2020). Interneuron types as Attractors and controllers. Annu. Rev. Neurosci..

[bib11] Fueta Y., Kunugita N., Schwarz W. (2005). Antiepileptic action induced by a combination of vigabatrin and tiagabine. Neuroscience.

[bib12] Grønborg M., Pavlos N.J., Brunk I., Chua J.J.E., Munster-Wandowski A., Riedel D., Ahnert-Hilger G., Urlaub H., Jahn R. (2010). Quantitative comparison of glutamatergic and GABAergic synaptic vesicles unveils selectivity for few proteins including MAL2, a novel synaptic vesicle protein. J. Neurosci..

[bib13] Krnjevic K., Schwartz S. (1967). The action of y-aminobutyric acid on cortical neurones. Exp. Brain Res..

[bib14] Kyoung M., Zhang Y., Diao J., Chu S., Brunger A.T. (2013). Studying calcium-triggered vesicle fusion in a single vesicle-vesicle content and lipid-mixing system. Nat. Protoc..

[bib15] Lai Y., Choi U.B., Leitz J., Rhee H.J., Lee C., Altas B., Zhao M., Pfuetzner R.A., Wang A.L., Brose N., Rhee J.S., Brunger A.T. (2017). Molecular mechanisms of synaptic vesicle priming by Munc13 and Munc18. Neuron.

[bib39] Leitz J., Wang C., Esquivies L., Peters J.J., Gopal N., Pfuetzner R.A., Wang A.L., Brunger A.T. (2024). Improved methods to isolate, purify, and functionally test specific synaptic vesicles. Nature Protocols.

[bib40] Leitz J., Wang C., Esquivies L., Pfuetzner R.A., Peters J.J., Couoh-Cardel S., Wang A.L., Brunger A.T. (2024). Beyond the MUN domain, Munc13 controls priming and depriming of synaptic vesicles. Cell Reports.

[bib16] Lai Y., Diao J., Cipriano D.J., Zhang Y., Pfuetzner R.A., Padolina M.S., Brunger A.T. (2014). Complexin inhibits spontaneous release and synchronizes Ca2+-triggered synaptic vesicle fusion by distinct mechanisms. Elife.

[bib38] Li X., Mooney P., Zheng S., Booth C.R., Braunfeld M.B., Gubbens S., Agard D.A., Cheng Y. (2013). Electron counting and beam-induced motion correction enable near-atomic-resolution single-particle cryo-EM. Nat. Methods.

[bib19] Loh K.H., Stawski P.S., Draycott A.S., Udeshi N.D., Lehrman E.K., Wilton D.K., Svinkina T., Deerinck T.J., Ellisman M.H., Stevens B., Carr S.A., Ting A.Y. (2016). Proteomic analysis of unbounded cellular compartments: synaptic clefts. Cell.

[bib20] Luscher B., Shen Q., Sahir N. (2011). The GABAergic deficit hypothesis of major depressive disorder. Mol. Psychiatry.

[bib37] Mastronarde D.N. (2005). Automated electron microscope tomography using robust prediction of specimen movements. J. Struct. Biol..

[bib21] Nuss P. (2015). Anxiety disorders and GABA neurotransmission: a disturbance of modulation. Neuropsychiatr. Dis. Treat..

[bib22] Obata K., Ito M., Sato N. (1967). Pharmacological properties of the postsynaptic inhibition by Purkinje cell axons and the action of 7-aminobutyric acid on deiters neurones. Exp. Brain Res..

[bib23] Petroff O.A.C. (2002). GABA and glutamate in the human brain. Neurosci. Rev. J. Bringing Neurobiol. Neurol. Psychiatry.

[bib24] Rebola N., Reva M., Kirizs T., Szoboszlay M., Lőrincz A., Moneron G., Nusser Z., DiGregorio D.A. (2019). Distinct nanoscale calcium channel and synaptic vesicle topographies contribute to the diversity of synaptic function. Neuron.

[bib25] Rizo J., Xu J. (2015). The synaptic vesicle release machinery. Annu. Rev. Biophys..

[bib26] Rothman J.E. (2014). The principle of membrane fusion in the cell (Nobel Lecture). Angew. Chem. Int. Ed..

[bib27] Sears S.M., Hewett S.J. (2021). Influence of glutamate and GABA transport on brain excitatory/inhibitory balance. Exp. Biol. Med..

[bib28] Südhof T.C. (2013). Neurotransmitter release: the last millisecond in the life of a synaptic vesicle. Neuron.

[bib29] Takamori S., Holt M., Stenius K., Lemke E.A., Grønborg M., Riedel D., Urlaub H., Schenck S., Brügger B., Ringler P., Müller S.A., Rammner B., Gräter F., Hub J.S., De Groot B.L., Mieskes G., Moriyama Y., Klingauf J., Grubmüller H., Heuser J., Wieland F., Jahn R. (2006). Molecular anatomy of a trafficking organelle. Cell.

[bib30] Takamori S., Riedel D., Jahn R. (2000). Immunoisolation of GABA-specific synaptic vesicles defines a functionally distinct subset of synaptic vesicles. J. Neurosci. Off. J. Soc. Neurosci..

[bib31] Tang X., Jaenisch R., Sur M. (2021). The role of GABAergic signalling in neurodevelopmental disorders. Nat. Rev. Neurosci..

[bib32] Tao C.-L., Liu Y.-T., Sun R., Zhang B., Qi L., Shivakoti S., Tian C.-L., Zhang P., Lau P.-M., Zhou Z.H., Bi G.-Q. (2018). Differentiation and characterization of excitatory and inhibitory synapses by cryo-electron tomography and correlative microscopy. J. Neurosci. Off. J. Soc. Neurosci..

[bib33] Tremblay R., Lee S., Rudy B. (2016). GABAergic interneurons in the neocortex: from cellular properties to circuits. Neuron.

[bib34] Trimmer J.S. (2020). Recombinant antibodies in basic neuroscience research. Curr. Protoc. Neurosci..

[bib35] Upmanyu N., Jin J., Emde H. von der, Ganzella M., Bösche L., Malviya V.N., Zhuleku E., Politi A.Z., Ninov M., Silbern I., Leutenegger M., Urlaub H., Riedel D., Preobraschenski J., Milosevic I., Hell S.W., Jahn R., Sambandan S. (2022). Colocalization of different neurotransmitter transporters on synaptic vesicles is sparse except for VGLUT1 and ZnT3. Neuron.

[bib36] Van Liefferinge J., Massie A., Portelli J., Di Giovanni G., Smolders I. (2013). Are vesicular neurotransmitter transporters potential treatment targets for temporal lobe epilepsy?. Front. Cell. Neurosci..

