# Supplementary material for: A new method for isolation and purification of fusion-competent inhibitory synaptic vesicles
Source: Curr Res Physiol. 2024 Feb 23;7:100121. doi: 10.1016/j.crphys.2024.100121 (PMC10990708; doi:10.1016/j.crphys.2024.100121)
Supplement: Supplementary data files [file mmc1.pdf]

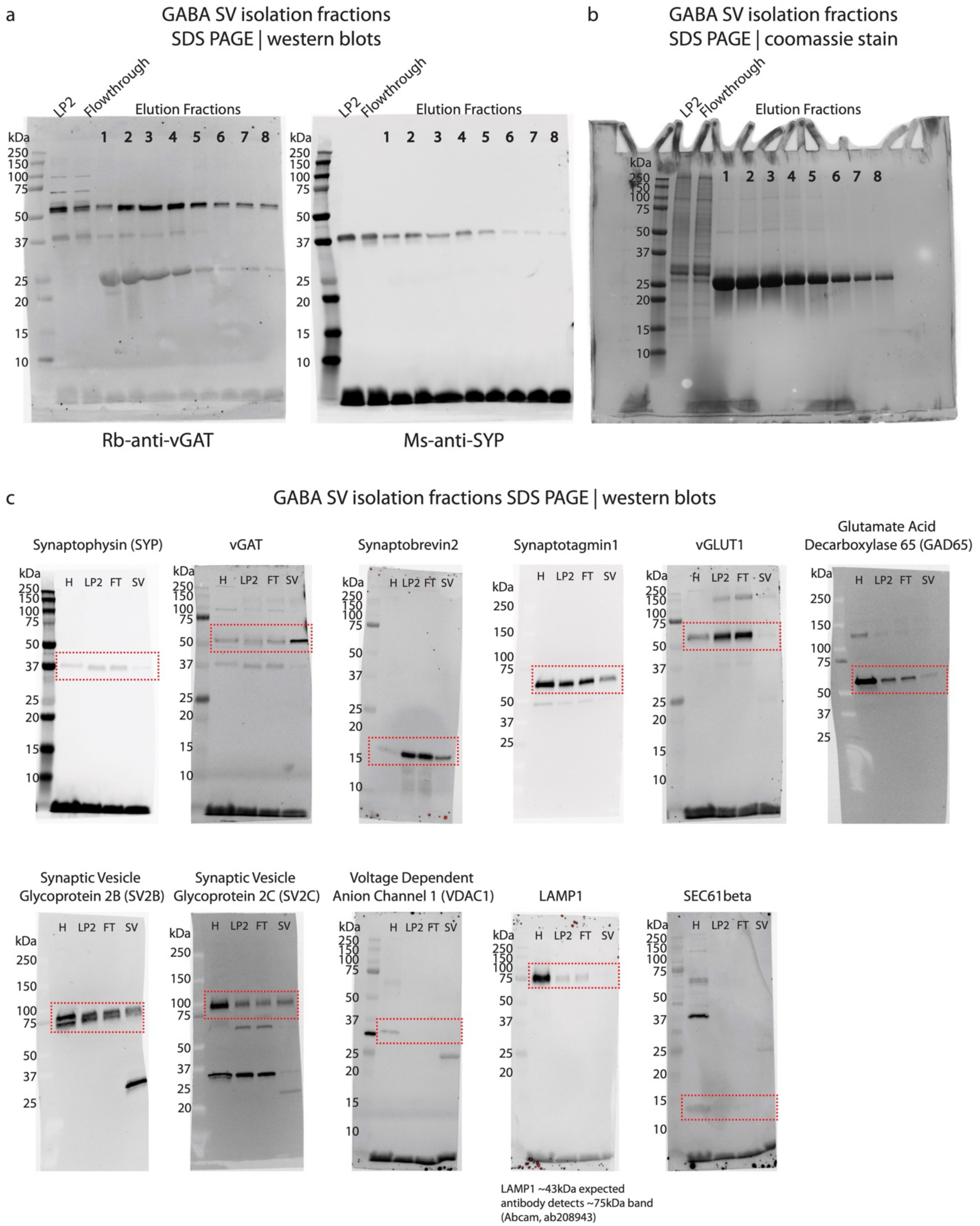

Supplementary Figure 1. Original western blot images corresponding to Figures 1 and 2, unadjusted, uncropped, and unrotated. (a) Western blot analysis of GABA SV isolation fractions including starting material (LP2) and flowthrough as well as biotin elution fractions 1-8. The membrane was blotted with a polyclonal rabbit anti-vGAT

antibody and an 800CW goat-anti-rabbit secondary antibody (**Supplementary Table 1** for antibody details). **(b)** Coomassie-stained SDS PAGE analysis of GABA SV isolation fractions including starting material, Streptactin flowthrough as well as biotin elution fractions 1-8. **(c)** Uncropped, original western blots of homogenate, LP2, FT, and purified SVs blotted for a variety of proteins (**Supplementary Table 1** for antibody details).

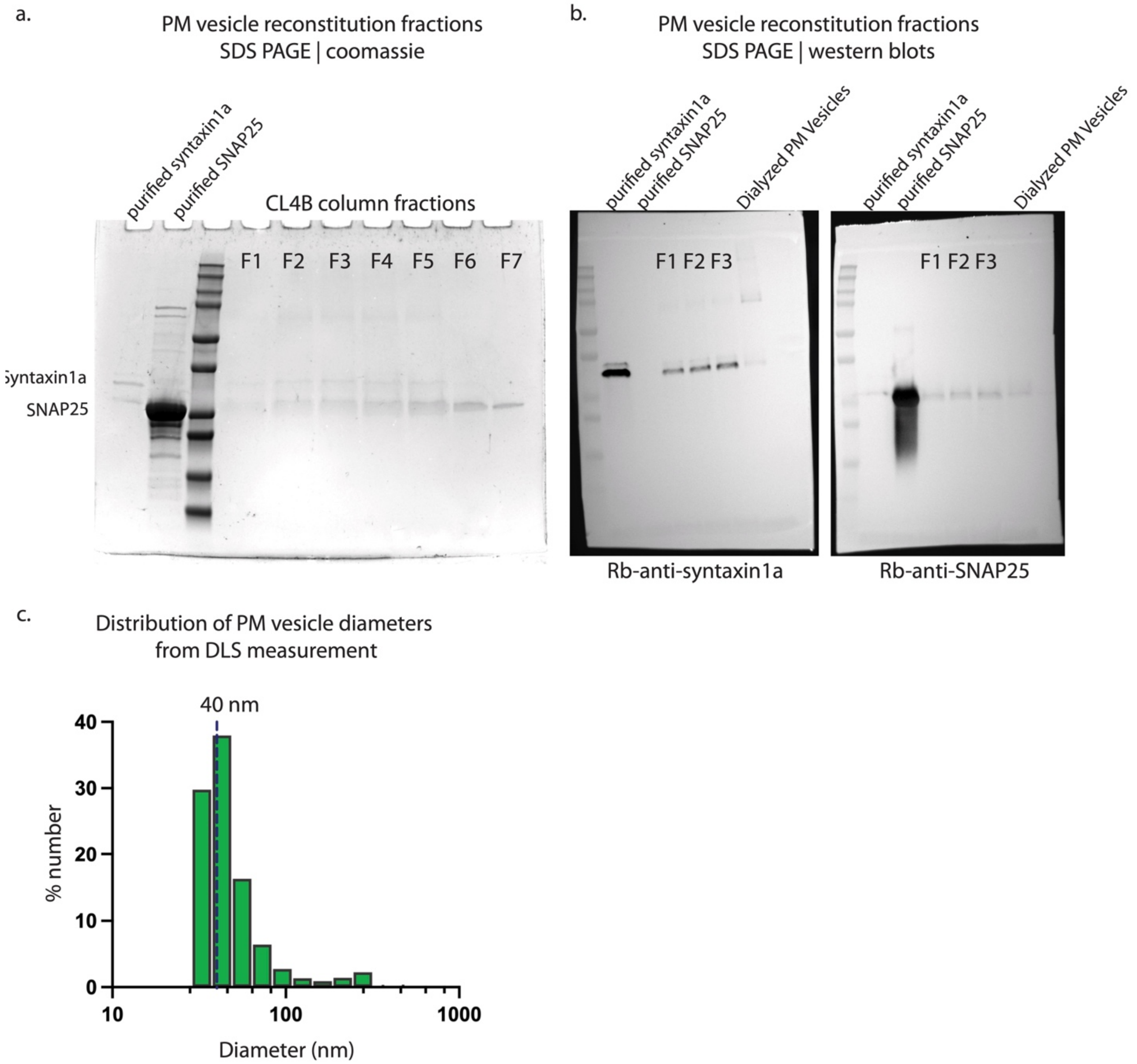

Supplementary Figure 2. Reconstitution of PM vesicles. (a) Coomassie-stained SDS PAGE image of PM vesicle reconstitution fractions, showing input protein as well as 200 µL fractions 1-4 after passing the protein-lipid mixture through a CL4B column. **(b)** Western blot images of PM vesicle reconstitution fractions showing input proteins and post CL4B fractions 1-3, as well as the PM vesicle sample after overnight dialysis. The top image is an anti-syntaxin western blot and the bottom image is an anti-SNAP 25 western blot. **(c)** Frequency distribution of PM vesicle diameters, as determined by dynamic light scattering measurements.

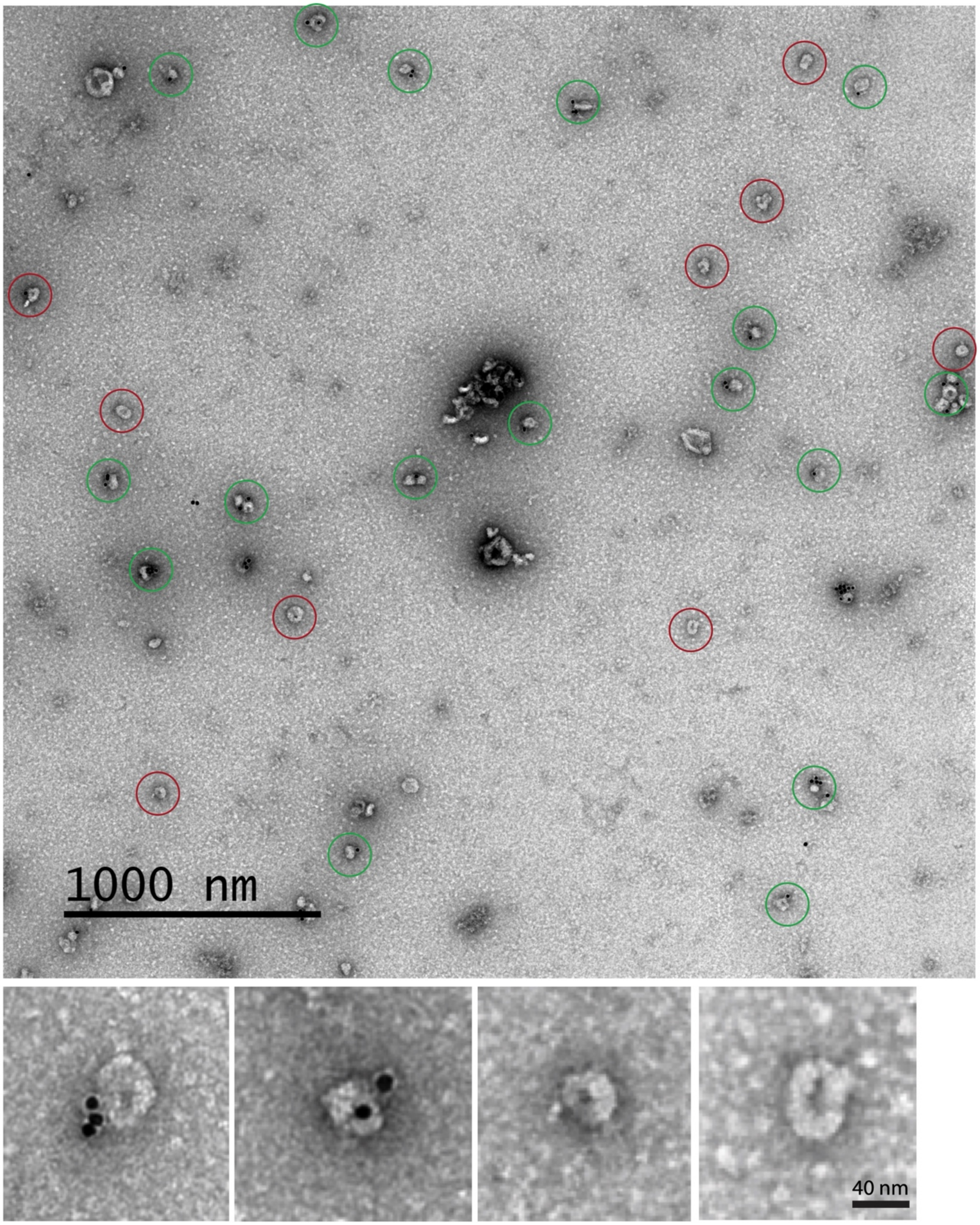

Supplementary Figure 3. Labeling of GABA SVs with anti-SYP-Alexa-647.
Representative negative stain EM micrograph of Alexa-647-SVs labeled with 15 nm gold conjugated anti-rabbit Fab. The dataset that includes this representative micrograph had a labeling efficiency between 40-60% and a range of partial efficiencies was observed for other preparations. Green circles denote SVs with gold particles

attached and red circles denote SVs without gold particles attached. Bottom: close-up view examples of GABA SVs with and without gold particles. Additional negative stain EM images are available in the data repository associated with this work.

Supplementary Table 1

Antibodies Used			
Antibody	Dilution Used	Vendor	Cat #
Rabbit - anti vGAT	1:2500	Synaptic Systems	131103
Mouse - anti Synaptophysin	1:500	Abcam	ab8049
Rabbit - anti vGLUT11	1:2500	Synaptic Systems	135303
Rabbit - Synaptotagmin1	1:1000	Abcam	ab126253
Mouse - Synaptobrevin/VAMP2	1:1000		
Rabbit - SV2C	1:1000	Synaptic Systems	119203
Rabbit - SV2B	1:1000	Synaptic Systems	119103
Rabbit - GAD65		Abcam	ab239372
Mouse - VDAC	1:1000	Santa Cruz Bio Technology	sc-390996
Rabbit - Sec61β	1:1000	Abcam	ab229542
Rabbit - LAMP1	1:1000	Abcam	ab208943
IRDye 800CW Goat anti-Rabbit IgG Secondary Antibody	1:5000	Licor	926-32211
IRDye 800CW Goat anti-Mouse IgG Secondary Antibody	1:5000	Licor	926-32210
IRDye 680CW Goat anti-Mouse IgG Secondary Antibody	1:5000	Licor	926-68070

Supplementary Table 2

Association Data	
Movies	25
Experimental preparations<br>(# of separate LP2 preparation)	1
Total ROI	9,728
Single vesicle association	296
Multiple vesicle association	17
Fusion Events	3
Immediate Fusion	1
Delayed Fusion	2

Supplementary Table 3

Triggered Fusion Data					
	Movies	Experimental preparations (# of separate LP2 preparation)	Total ROI	Fusion Events	Fusion events during first second after Ca/Mg entry
500 μM Ca^{2+}	62	3	25237	135	23
250 μM Ca^{2+}	28	3	11,849	65	13
50 μM Ca^{2+}	13	3	3,537	41	2
500 μM Mg^{2+}	27	3	12951	13	0
